# Draft Genome Sequences of Five Vibrio neptunius Strains Isolated from Hatcheries of Bivalve Mollusks

**DOI:** 10.1128/MRA.00237-21

**Published:** 2021-04-29

**Authors:** Fabian Galvis, Susana Prado, Juan L. Barja, Manuel L. Lemos, Miguel Balado

**Affiliations:** a Departamento de Microbiología y Parasitología, Instituto de Acuicultura y CIBUS-Facultad de Biología, Universidade de Santiago de Compostela, A Coruña, Spain; University of Southern California

## Abstract

Vibrio neptunius is a Gram-negative bacterium that has been shown to cause disease in marine bivalve mollusk larvae. Here, we report the draft genome sequences and annotations of five *V. neptunius* strains isolated from larvae of European oyster (Ostrea edulis) and Manila clam (Ruditapes philippinarum) at hatcheries in Galicia, northwest Spain.

## ANNOUNCEMENT

The global production of marine bivalves for human consumption is more than 15 million tonnes per year, which is about 14% of the total marine production in the world (1–3). The decrease in natural beds has led to the need to establish hatcheries to provide farms with juveniles. The optimum conditions for the growth and development of bivalve larvae in hatcheries (e.g., densities, temperature, and organic matter load) increase the growth and multiplication of bacteria and the accumulation of their metabolites, which are associated with the decrease of the growth and mortality in larvae and juveniles of many bivalve mollusks (4, 5). Vibriosis, which is caused by some *Vibrio* spp., is the main bottleneck of the production process in bivalve hatcheries, leading to high larval mortality rates and loss of production lots. *V. neptunius* was identified as a pathogen responsible for larval and spat mortality episodes in clam and oyster cultures, causing important economic losses for mollusk hatcheries (6).

The 5 *V. neptunius* strains sequenced here were isolated from vibriosis outbreaks affecting bivalve hatcheries in Galicia, northwest Spain. Identification was based on phenotypic and genetic characterization by using conventional biochemical tests, the API 20E system (bioMérieux), and 16S rRNA gene sequencing. All details on the isolation of the strains, phenotypic characterization process, and 16S rRNA gene sequencing were described by Prado et al. (5). *V. neptunius* strains PP-145.98, PP-256, and PP-259 were isolated from clam larvae (Ruditapes philippinarum), and strains PP-307 and PP-313 were isolated from oyster larvae (Ostrea edulis).

Genomic DNA was extracted from cultures of *V. neptunius* that had been grown overnight at 25°C, with shaking, in Trypticase soy broth supplemented with 1% NaCl, using the EasyDNA genomic DNA purification kit (Invitrogen). Sequencing was performed by the Fisabio Public Health Sequencing and Bioinformatics Service (Valencia, Spain), using 300-bp paired-end libraries prepared with the Illumina Nextera XT kit, and ran on an Illumina MiSeq system. The reads were inspected for data quality using FastQC version 0.11.5 (http://www.bioinformatics.babraham.ac.uk/projects/fastqc). Default parameters were used for all software unless otherwise noted. Reads were quality trimmed using PRINSEQ v. 0.20.4 (7), clipping the read when the quality score of a 3-bp sliding window was below 20 and omitting reads of <100 bp. Reads were assembled using SPAdes v. 3.11.1 1 (-careful; -mismatch-correction; -k 21, 33, 55, 77, 99, 127 bp) (8). Scaffolds lower than 200 bp or with low coverage were removed. Gene predictions and annotations were performed by the National Center for Biotechnology Information (NCBI) through the Prokaryotic Genome Annotation Pipeline (PGAP) v. 5.1 (9). An online software GC content calculator (https://www.sciencebuddies.org/science-fair-projects/references/genomics-g-c-content-calculator) was used to calculate the GC content of the genomes. Further information on the genome parameters is given in Table 1.

**TABLE 1:** Sequencing results and annotation of *V. neptunius* strains isolated from oyster and clam larvae

Characteristic	Data for strain:				
	PP-145.98	PP-256	PP-259	PP-307	PP-313
Raw sequencing results
Total no. of reads	2,607,130	3,517,632	3,895,514	2,228,868	3,364,076
Total bases (Mbp)	611.6	821.9	913.6	525.5	786.6
Avg read length (bp)	233.5	232.6	234	234.8	232.9
Avg coverage depth (×)	104.8	141.3	160.4	90.4	135.1
Assembly results
No. of contigs	169	178	151	186	171
Genome size (bp)	5,275,089	5,276,515	5,147,033	5,276,531	5,273,268
*N*_50_ (bp)	129,442	129,040	188,696	138,645	129,198
GC content (%)	45.1	45.1	45.2	45.1	45.1
Annotation results
No. of CDS^*a*^	4,795	4,809	4,670	4,819	4,799
No. of genes	4,920	4,936	4,802	4,942	4,926
No. of RNA genes	125	127	132	127	127
No. of rRNAs	16	16	26	19	16
No. of tRNAs	105	107	102	104	107
Accession no.
BioSample	SAMN18024316	SAMN18024317	SAMN18024318	SAMN18024319	SAMN18024320
SRA	SRR14089945	SRR14089942	SRR14089941	SRR14089943	SRR14089944
GenBank	JAFHLB010000000	JAFHLC000000000	JAFHLD000000000	JAFHLE000000000	JAFHLF000000000

a CDS, coding DNA sequences.

The sequencing of the genomes of pathogenic strains of *V. neptunius* would allow the identification and characterization of sequences related to possible virulence factors.

### Data availability.

These nucleotide sequences have been deposited in DDBJ/ENA/GenBank as BioProject PRJNA703775 under the accession numbers provided in Table 1.
